# Precision Medicine

**DOI:** 10.1590/S1679-45082017ED4016

**Published:** 2017

**Authors:** João Renato Rebello Pinho

**Affiliations:** 1 Hospital Israelita Albert Einstein, São Paulo, SP, Brazil;; Faculdade de Medicina, Universidade de São Paulo, São Paulo, SP, Brazil.

Precision Medicine, Personalized Medicine, and Individualized Medicine (and even older terms, such as pharmacogenomics or pharmacogenetics) have often been used over the last years to mean the use of biological markers (obtained by ordering a few supplementary tests by physicians) that may indicate particular genetic characteristics of the different affected individuals and of the various diseases that affect them (such as genetic, oncologic, or infectious diseases). These biomarkers may be used to indicate the most adequate treatment and follow-up for these disorders, which can be different for each individual. The term ‘Precision Medicine’ is currently preferred, since besides Genetics, it also involves lifestyle and environmental characteristics^1^

It is implicit in this definition that the methods used for the diagnosis necessarily went through a significant progression, allowing the definition of specific characteristics of these individuals and their diseases, which could not have been imagined few years ago. In parallel, therapeutic advances have also been relevant over the last decades, when knowledge of Genetic Engineering enabled us to obtain, on a large scale, different compounds used in ever more specific ways in several conditions, with variable effects according to the genetic profile.

This topic has been increasingly discussed by the medical community over these last years. Many scientific events on the theme have been held all over the world to discuss the use of new biomarkers, and to improve diagnosis and treatment of diverse diseases. For example, *Hospital Israelita Albert Einstein* has hold an International Symposium on Precision Medicine (previously called International Symposium on Personalized Medicine) since 2013. At this event, the main advances in the area are discussed, that is, how these biomarkers are used in various fields of medicine. A review of this topic was recently published by this journal.^2^

Scientific articles, and specific meetings held by various national and international scientific societies, currently discuss the importance of this area, in which the “omics” are the subjects of many events. With “omics”, we address the sciences that encompass knowledge of the human genome and its products, and interrelations, which are areas that initiate with the basic sciences, but whose results can already be put into practice today in patient follow-up. Thus, within this field, there is genomics and its variations, such as epigenomics (study of epigenetic alterations, such as interactions of DNA with histones and methylations of nucleotides, which can change the expression of cell genes) and transcriptomics (study of RNAs expressed by the cells),^3^ or as studies with specific applications, such as the already mentioned pharmacogenomics and nutrigenomics (for studies about interactions of food with the genome).^4^ Microbiomics is the study of the microorganisms present in different body composites, especially within the gastrointestinal tract, but in other locations as well, including the urinary tract, eyes, skin, etc.^5,6^ There are also studies that involve the composition of other macromolecules, such as lipidomic and proteomic. Further, there are studies of metabolic products, such as metabolomics, that can also signal the presence of specific diseases.^3^

The first applications described initiated in pharmacogenomic studies, in which mapping of the variants of some important genes for drug metabolism showed why different doses of drugs had different effects, according to the genetic profile of the genes involved in their metabolism. For some medications, genetic variants can cause significant side effects that their dosage needs to be adjusted or the drug must be avoided in some patients.^2^

The effect of some drugs used to treat various infectious diseases may be very different according to the genetic profile of patients. The most traditional example of this is therapy with interferon and ribavirine, used for hepatitis C, which is much more effective in patients with polymorphism CC near the interleukin 28 gene (which now was located in the middle of an intron of the interferon lambda-4 gene, later discovered).^7^Currently, with the use of new forms of treatment for hepatitis C, using direct antiviral agents, this marker has lost its importance, but some are starting to discuss the relevance of verifying the presence of resistance to these agents, especially the NS5A inhibitors in treating patients with such a class of drugs.^8^ For the treatment of other viral diseases, such as aids, detection of specific mutations of resistance to the antivirals of the viral genome have their use established.^9^ In this case, the presence of some particular types of polymorphisms in genes involved in drugs metabolism of or in the major histocompatibility complex is important in the determination of the best treatment to be used.^10^

Nevertheless, the area in which Precision Medicine has shown itself most developments is Oncology. Different genetic tests are used as markers that indicate potential therapeutic agents for different neoplasms in various tissues. Detection of HER-2 by immunohistochemical technique indicating the treatment of breast cancer with trastuzumab was one of the first precision medicine markers, but in the area of Oncology, there are various assays that could indicate that some medications are effective (*e.g*., the presence of the BCR/ABL or PML/RARA translocation, indicating specific treatments for leukemias, or the presence of mutation V600E, indicating specific treatment in melanomas).^2^

Even more sophisticated tests using next generation sequencing techniques allow an analysis of several genes simultaneously, enabling a better opportunity to find appropriate medications for neoplasms. New tests detect not only specific punctual mutations in different genes, but also large deletions and insertions, and yet other types of more recently described mutations are under study − and are more important, especially for immunotherapy of neoplasms.^11^

The detection of circulating DNA derived from the tumor also opens up the possibility of monitoring the presence of these markers. They indicate the best possible treatments and following up the modifications in the molecular profile of lesions, without the need to access the tumor tissue, whether by detecting free circulating DNA (cfDNA) or circulating tumor cells (ctDNA).^12^ Indeed, there are currently approved diagnostic systems for the detection of some of these markers, such as mutations in the Epidermal Growth Factor Receptor (EGFR). Its detection in peripheral blood has already been approved by the Food and Drug Administration (FDA) for lung cancer, as well detection systems for various other markers simultaneously.^13^

In addition to the area of infectious and oncologic diseases, metabolic diseases also start to be targeted by these studies, and it is possible that genetic tests will be developed for this purpose in the near future to evaluate the risk of type 2 diabetes^14^ or of cerebrovascular diseases,^15^ including with the evaluation of the microbioma.^16^

Precision Medicine is not restricted only to molecular markers. In reality, the use of tests in any area of Medicine can be associated with its definition, as long as it is a supplementary test with an important role in the differentiation between a treatment specific for a disease that affects a given individual, such as imaging tests: the emerging discipline of radiogenomics links genotype information to the fenotypic disease found in the medical imaging exams.^17^

In Medicine, progresses are always previewed and possibly, the greatest ones come from methodologies that allowed us to manipulate the genetic material, which would be used in the future. Some ethical and biosafety issues still need to be discussed before the routine implementation of this treatment. However, the discovery of techniques enabling direct and targeted manipulation of genes potentially involved with the development of various diseases, particularly oncological conditions, have already begun to be used even in some countries that take greater care to allow the use new methodologies.^18^

This type of progress for treatment and diagnosis has already begun and should advance in the next few years. The changes in the area of Sciences have occurred very rapidly, as well as the transfer of many of these developments to medical areas. The constant progress in information technology enables a large quantity of data to be integrated as never done before, which allows better knowledge of the individual, disease, population group, environment, eating habits, and life conditions.^19^ The integration of these data, in a broader way generates deeper knowledge about human health for the individual and for population groups in different diseases. This is another change that will allow the application of an even more precise medicine.
